# EGb761, a Ginkgo Biloba Extract, Is Effective Against Atherosclerosis In Vitro, and in a Rat Model of Type 2 Diabetes

**DOI:** 10.1371/journal.pone.0020301

**Published:** 2011-06-02

**Authors:** Soo Lim, Ji Won Yoon, Seon Mee Kang, Sung Hee Choi, Bong Jun Cho, Min Kim, Ho Seon Park, Hyun Ju Cho, Hayley Shin, Young-Bum Kim, Hyo Soo Kim, Hak Chul Jang, Kyong Soo Park

**Affiliations:** 1 Department of Internal Medicine, Seoul National University Bundang Hospital, Seongnam, Korea; 2 Department of Internal Medicine, Seoul National University College of Medicine, Seoul National University Hospital, Seoul, Korea; 3 Johns Hopkins Bloomberg School of Public Health, Baltimore, Maryland, United States of America; 4 Division of Endocrinology, Diabetes and Metabolism, Department of Medicine, Beth Israel Deaconess Medical Center and Harvard Medical School, Boston, Massachusetts, United States of America; Pennington Biomedical Research Center, United States of America

## Abstract

**Background:**

EGb761, a standardized *Ginkgo biloba* extract, has antioxidant and antiplatelet aggregation and thus might protect against atherosclerosis. However, molecular and functional properties of EGb761 and its major subcomponents have not been well characterized. We investigated the effect of EGb761 and its major subcomponents (bilobalide, kaemferol, and quercetin) on preventing atherosclerosis *in vitro,* and in a rat model of type 2 diabetes.

**Methods and Results:**

EGb761 (100 and 200 mg/kg) or normal saline (control) were administered to Otsuka Long-Evans Tokushima Fatty rats, an obese insulin-resistant rat model, for 6 weeks (from 3 weeks before to 3 weeks after carotid artery injury). Immunohistochemical staining was performed to investigate cell proliferation and apoptosis in the injured arteries. Cell migration, caspase-3 activity and DNA fragmentation, monocyte adhesion, and ICAM-1/VCAM-1 levels were explored *in vitro*. Treatment with EGb761 dose-dependently reduced intima-media ratio, proliferation of vascular smooth muscle cells (VSMCs) and induced greater apoptosis than the controls. Proliferation and migration of VSMCs *in vitro* were also decreased by the treatment of EGb761. Glucose homeostasis and circulating adiponectin levels were improved, and plasma hsCRP concentrations were decreased in the treatment groups. Caspase-3 activity and DNA fragmentation increased while monocyte adhesion and ICAM-1/VCAM-1 levels decreased significantly. Among subcomponents of EGb761, kaemferol and quercetin reduced VSMC migration and increased caspase activity.

**Conclusions:**

EGb761 has a protective role in the development of atherosclerosis and is a potential therapeutic agent for preventing atherosclerosis.

## Introduction

*Ginkgo biloba L.* (Ginkgoaceae), known as the ‘maidenhair tree’, is the best-selling herbal remedy in the USA [1]. Traditionally, the fruits and seeds of Ginkgo have been used in Oriental medicine to improve chronic cough or enuresis [2]. Since the early 1990s, EGb761, a standardized extract of Ginkgo leaves, has become the most popularly used dietary supplement for treating vascular circulation problems and improving memory.

There are two major fractions in this extract: flavonoids and terpenes. Interestingly, these two have different properties which are responsible for exerting unique and diverse pharmacological actions of EGb761. The flavonoid fraction has antioxidant effects resulting from direct attenuation of reactive oxygen species by chelating pro-oxidant transitional metal ions, and also by promoting the expression of antioxidant proteins which in turn increases antioxidant metabolites such as glutathione [3]–[5]. The chemical structure of flavonoids comprising of an aromatic ring and a double bond seem to react preferentially with hydroxyl radicals [2]. The terpene lactones include the ginkgolides A, B, C, J and M, and bilobalide [6]. These were found to reduce platelet activation and aggregation by antagonizing platelet activating factor [7], [8]. This gives EGb761 the potential to improve blood circulation. In addition, bilobalide, a sesquiterpene trilactone, was shown to reduce cerebral edema, cortical infarct volume and ischemic damage in patients following a stroke [9].

EGb761 has also been shown to have various antiapoptotic properties[6] and to inhibit amyloid-beta aggregation [10]. Therefore, it has been used to improve cardiovascular and peripheral vascular insufficiency, to protect against neurological disorders such as ischemic injury and to treat cerebral disorders such as cognitive decline and memory impairment [9].

Interestingly, in addition to its neurological and vascular protective effects, EGb761 has been reported to reduce hyperglycemia. Rapin et al. reported that EGb761 increased glucose uptake and glycogen synthesis, and Tanaka et al. showed that the glucose-lowering effect of Ginkgo extracts was caused by the inhibition of alpha-amylase and glucosidase [11], [12].

Although EGb761 has beneficial effects on blood circulation and hyperglycemia in patients with diabetes, direct studies on its effects against atherosclerosis are limited. Therefore, we investigated the protective effect of EGb761 on atherosclerosis in a rat model of obese type 2 diabetes. We also examined the possible role of EGb761 and its major subcomponents on the development and progression of atherosclerosis *in vitro*. In addition, the effects of EGb761 on glucose homeostasis, circulating adipocytokines and inflammatory markers were evaluated.

## Materials and Methods

### Animal and Material

Thirty-six 5-week-old male Otsuka Long-Evans Tokushima Fatty (OLETF) rats were donated by the Otsuka Pharmaceutical Co. (Tokushima, Japan). They were allowed to grow to 24 weeks of age, when obesity and insulin resistance develop. The OLETF rats were held in the Preclinical Laboratory of Seoul National University Bundang Hospital, South Korea, for the study duration. All animals were handled in compliance with the Guide for Experimental Animal Research of the Laboratory, Seoul National University Bundang Hospital. Seoul National University Bundang Hospital Ethics Committee for Animal Study approved this study (06-2008-096).

We divided the rats into three groups (n = 12 each) and treated them as follows: controls (5 ml normal saline per day), rats given 100 mg/kg of EGb761 per day (EGb100), and rats given 200 mg/kg of EGb761 per day (EGb200). All rats were fed a regular chow diet and had free access to water. EGb761 or normal saline were administered using an oral Zonde needle (Natsume, Tokyo, Japan) at 9–10 am for 6 weeks (from 3 weeks before to 3 weeks after balloon injury). EGb761 was supplied by Dr. Willmar Schwabe Pharmaceuticals (KG, Karlsruhe, Germany). Two major ingredients were ginkgo flavone glycoside (24.31%) and terpene trilactones (5.42%). Subcompounds of EGb761 are described in Table S1.

Additional experiments were carried out involving male ApoE-/- mice (n = 12) 5 weeks of age, which were purchased from Jackson laboratories (Bar Harbor, Maine). For 2 months all mice were fed high fat cholesterol diet #88137 (Harlan-Teklad; 42% fat, 1.25% cholesterol) beginning at 5 weeks of age. After 2 months, mice were divided into three groups: control, EGb100 and EGb200 (n = 4 each) and continued on the high fat diet. Mice were sacrificed after 2 months post treatment to evaluate the degree of plaque formation. Whole aortas were opened lengthwise, fixed in 10% formalin, stained with oil red O and quantified by computerized morphometrics. The results for atherosclerotic plaque were expressed as the mean±standard error of the mean.

### Rat carotid artery balloon denudation injury and morphometric analysis

Balloon injury of the carotid artery was performed according to a well-established injury model [13]. The extent of neointimal formation in histologically stained sections was quantified by computed planimetry. The cross-sectional areas of the blood vessel layers (i.e., the lumen, intimal, and medial areas) were quantified in three different sections (proximal, middle, and distal) using Image-Pro Plus Analyzer version 4.5 (Media Cybernetics, Bethesda, MD, USA). The intima to media ratio (IMR) was calculated from the mean of these determinations.

### Immunohistochemical staining for proliferating cells and inflammatory cells

To detect proliferating cells, immunohistochemical staining for proliferating cell nuclear antigen (PCNA) was performed, as previously described [14]. The proliferation index was defined as the percentage of PCNA-positive cells relative to the total nucleated cell count in four different sectors per tissue section. Staining for the glycoprotein ED-1 (Dako, Carpinteria, CA, USA), which is expressed predominantly on the lysosomal membrane of tissue macrophages, was performed on the injured carotid arteries.

### Staining for Apoptosis

Detection of apoptotic cells in vivo was also performed using the terminal deoxynucleotidyl transferase-mediated dUTP nick and labeling (TUNEL) method with minor modification [15]. Briefly, 5 µm sections were deparaffinized and incubated with proteinase K (Dako) (20 µg/ml) for 15 min at room temperature. An apoptosis detection kit (Apoptag, Intergen Company, Purchase, NY) was used with the chromogen DAB. Sections were counterstained with Mayer's hematoxylin (Dako). Apoptotic cells were quantified by determining the percentages of TUNEL-positive cells among all nucleated cells counted in four different sectors per tissue section.

### Glucose Metabolism, Adipocytokine and Inflammatory Markers

Possible relevant factors affecting the degree of neointimal formation, such as glucose homeostasis, adipocytokines and inflammatory status were evaluated. To assess glucose homeostasis, an intraperitoneal glucose tolerance test (IPGTT) was performed at baseline and 6 weeks after the EGb761 treatment. Rat adiponectin, high-sensitivity C-reactive protein (hsCRP), monocyte chemoattractant protein-1 (MCP1), TNFα and plasminogen activator inhibitor-1 (PAI1) activity were measured using commercial kits.

### Cell Culture

Rat aortic smooth muscle cells (RAoSMCs; Bio-bud Seoul, Korea) and human umbilical vein endothelial cells (HUVECs; Cambrex, Walkersville, MD) were used for *in vitro* experiments. See the Table S1 and Figures S1, S2, S3, S4, S5, S6 for detailed information.

### Cell Proliferation and Cytotoxicity

Effects of EGb761 on RAoSMCs proliferation was determined by a modified 3-(4,5-dimethyl-thiazol-2-yl)-2,5-diphenyltetrazolium bromide (MTT) assay. To investigate cell viability of EGb761, calcein-acetoxymethyl ester (calcein-AM) cell viability assay kit was used (Biotium, Hayward, CA, USA).

### Cell Migration

RAoSMCs were grown to confluence in 6-well plates (SonicSeal Slide Wells, Nalge Nunc, Rochester, NY) and then starved in DMEM with 0.5% FBS for 48 h. Thereafter, each well was divided into a 2×3 grid. Using 100-1000 µL pipette tips, a linear wound was made in each hemisphere of the well. Immediately after this, the medium was replenished with starvation medium. TNFα (10 ng/ml) was mixed in starvation medium. Cells were allowed to migrate for 24 h at 37°C. Images were taken of the intersections of the linear wound and each grid line, which resulted in 3 fields per well.

### Monocyte Adhesion Assay

U937 cells (Human leukemic monocyte lymphoma cell line; Abcam, Cambridge, MA) were washed 3 times with serum-free RPMI medium and then resuspended in the same medium. The U937 cells (1.25×10^4^) were added to the HUVEC monolayers stimulated with TNFα (10 ng/ml) for 18 h and incubated for 30 min at 37°C under 5% CO_2_ in air. Unbound cells were removed by washing 3 times with PBS. EBM-2 medium was then added and all U937 cells adhering to an endothelial cell were counted in 3–5 randomly selected fields of view in each well using a phase-contrast microscope.

### Intercellular Adhesion Molecule (ICAM) and Vascular Cell Adhesion Molecule (VCAM)

For reverse transcription–polymerase chain reaction (RT-PCR), RNA was isolated from HUVECs according to the TRIZOL protocol (Gibco Life Technology). Primer and probe sequences for ICAM and VCAM were described previously [16]. The RT-PCR was performed with the TaqMan system (Prism 7700 Sequence Detection System, PE Biosystems, Foster City, CA). Thermal cycling conditions comprised an initial denaturation step at 94°C for 5 min, followed by 94°C for 30 sec, 58°C for 60 sec, and 72 for 30 sec for 40 cycles. For quantification, the target sequences were normalized in relation to the human GAPDH product (Clonetech, Heidelberg, Germany).

### Immunoblot Analysis for Caspase-3 Activity

Immunoblot analysis was performed with primary antibodies directed against pro-caspase-3; cleaved caspase-3; β-actin (all from Santa Cruz Biotechnology). The bands were visualized with enhanced chemiluminescence and quantified by densitometry. Cleaved caspase 3-expression data were normalized by β-actin levels.

### DNA fragmentation

DNA fragmentation, a distinctive feature of apoptosis at the biochemical level, was evaluated with EGb761 (100 and 200 µg/ml) and its subcomponents; kaemferol, quercetin and bilobalide (10, 20 and 50 µg/ml, respectively).

### Statistical Analysis

Results are reported as the mean±standard error (SE). Mean values were compared for the EGb761-treated groups and control group by ANOVA with a post hoc test and p*<*0.05 was considered statistically significant. Analysis was done using SPSS for Windows version 11.0 (SPSS Inc., Chicago, IL).

## Results

### In vivo Inhibition of Neointimal Formation and Plaque Development

**Figure 1:**
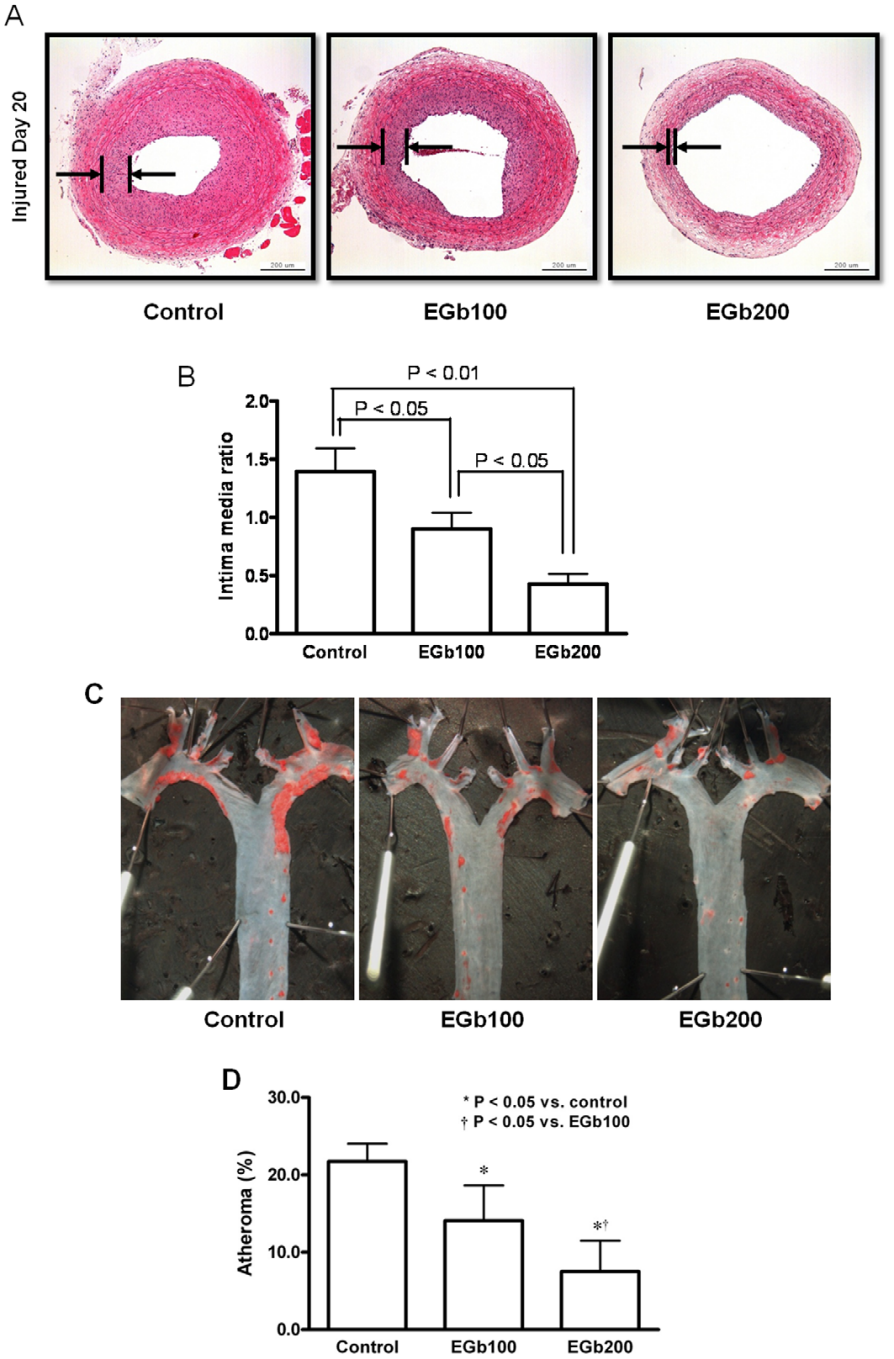
In vivo inhibition of neointimal formation after 6 weeks of treatment with EGb761. A. H&E stained sections of the three groups. B. Intima to media ratios (IMRs) in the three groups (n = 10 in each group). Treatment with EGb761 produced a lower IMR than controls in a dose-dependent manner (the lower IMR with the higher dose of EGb761, p<0.05). C. Representative examples of aortas from ApoE-/- mice stained en-face with Oil-Red. Red color indicates the aortic arch where plaque accumulation is the highest. D. Quantification of aortic arch plaque in the three groups expressed as the mean±SEM percent. A dose-dependent decreased plaque volume was found in the EGb groups.

Three weeks after injury, the EGb761-treated groups showed a significant reduction in neointimal formation compared to the control group (**Fig. 1A**). The EGb200 group showed less neointimal formation than the EGb100 group (**Fig. 1A, B**). As shown in **Fig. 1B**, there was a dose-dependent reduction of the IMR between the two EGb761-treated groups (Control, 1.40±0.20; EGb100, 0.90±0.14; EGb200, 0.43±0.09, p<0.05). Representative examples of aortas from ApoE-/- mice stained en-face with Oil-Red are shown in **Fig. 1C**. Red color indicates the aortic arch where plaque accumulation is the highest. **Fig. 1D** shows the quantification of aortic arch plaque in the three groups expressed as the mean±SEM percent. A dose-dependent decreased plaque volume was found in the EGb groups.

### Inhibited Proliferation and Sustained Apoptosis of Vascular Smooth Muscle Cells and Reduced Inflammatory Cells

**Figure 2:**
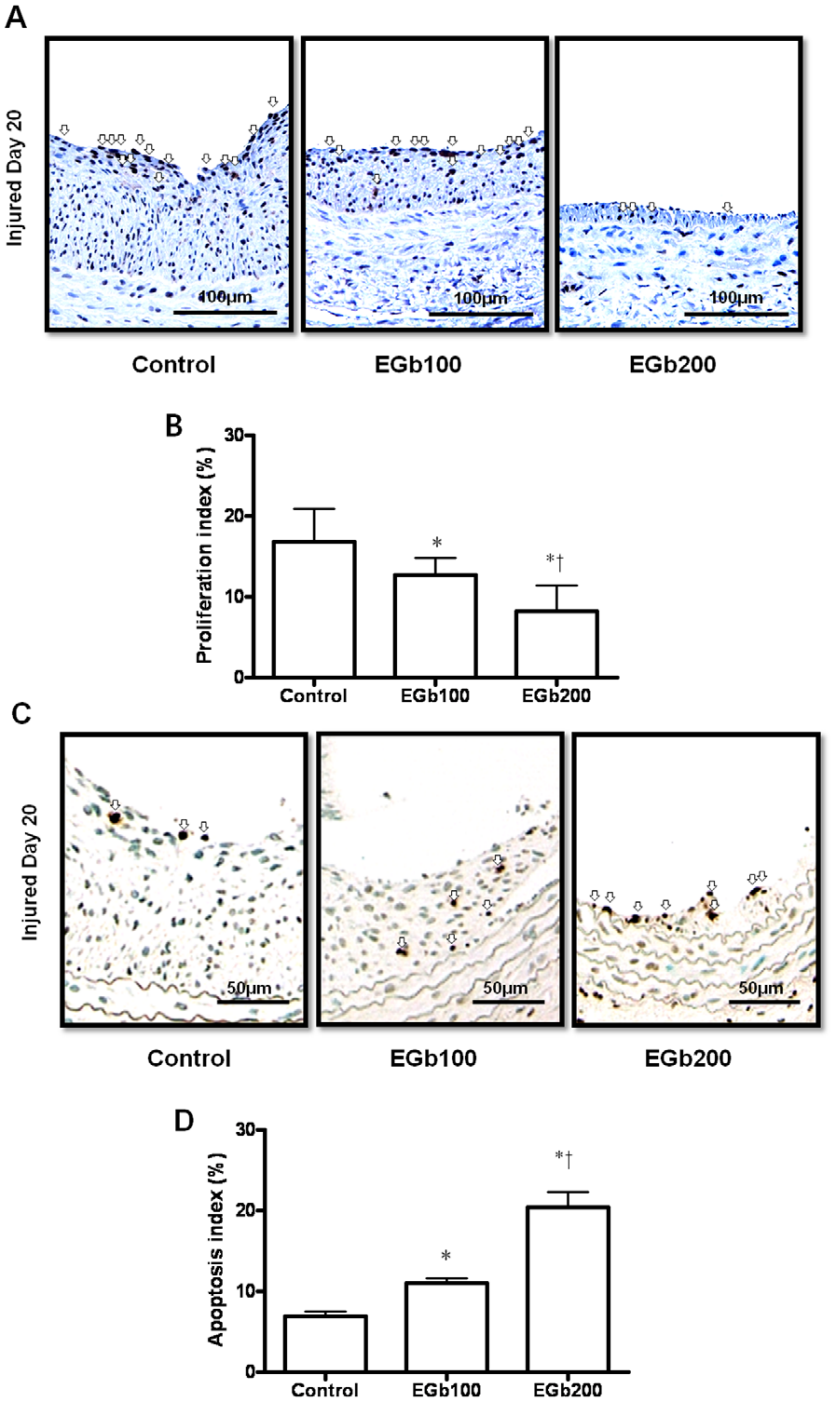
Effects of EGb761 (100 or 200 mg/kg) treatment or normal saline (control) on the proliferation and apoptosis of vascular smooth muscle cells. A. Cell proliferation measured by immunostaining for proliferating cell nuclear antigen (PCNA) was markedly lower in the EGb761-treated groups than in the controls (open arrow). B. The proliferation index was significantly lower in the EGb761-treated groups than in the control group. There was a dose-dependent pattern in the level of proliferation between EGb761-treated groups (* p<0.05 vs. control and † p<0.05 vs. EGb100). C. TUNEL staining of the three groups (open arrow). D. Apoptosis index (%) at 3 weeks after balloon injury. Apoptosis was significantly higher in the EGb761-treated groups than in the control group, and there was a dose-dependent pattern in the level of apoptosis between EGb761-treated groups (* p<0.05 vs. control and † p<0.05 vs. EGb100).

Cell proliferation and apoptosis are important contributors to neointimal formation after balloon injury. We performed experiments investigating the role of EGb761 in reducing proliferation and promoting apoptosis of cells. As shown in **Fig. 2A**, the proliferative index was significantly lower in the EGb761 treated groups than in the control (control, 16.8±4.1%; EGb100, 12.7±2.1%; EGb200, 8.2±3.2%, p<0.05 vs. control, respectively). There was a dose-dependent decrease in proliferation between the two EGb761-treated groups (p<0.05) (**Fig. 2B**). At 3 weeks after injury, the apoptosis index was significantly higher in the EGb761-treated groups than in the control group (**Fig. 2C**). There was also a dose-dependent increase in the level of apoptosis between the two EGb761 groups (control 6.9±0.6%; EGb100, 11.0±0.6%; EGb200, 20.4±1.9%, p<0.05) (**Fig. 2D**). Immunohistochemical staining for ED-1 (an inflammatory cell marker in the rat) in the injured carotid vessel wall also showed that ED-1 positive cells were more frequent in the control group than in the EGb761-treated groups (**Fig. S1**).

### Decrease in Proliferation and TNFα-directed Migration of RAoSMCs

**Figure 3:**
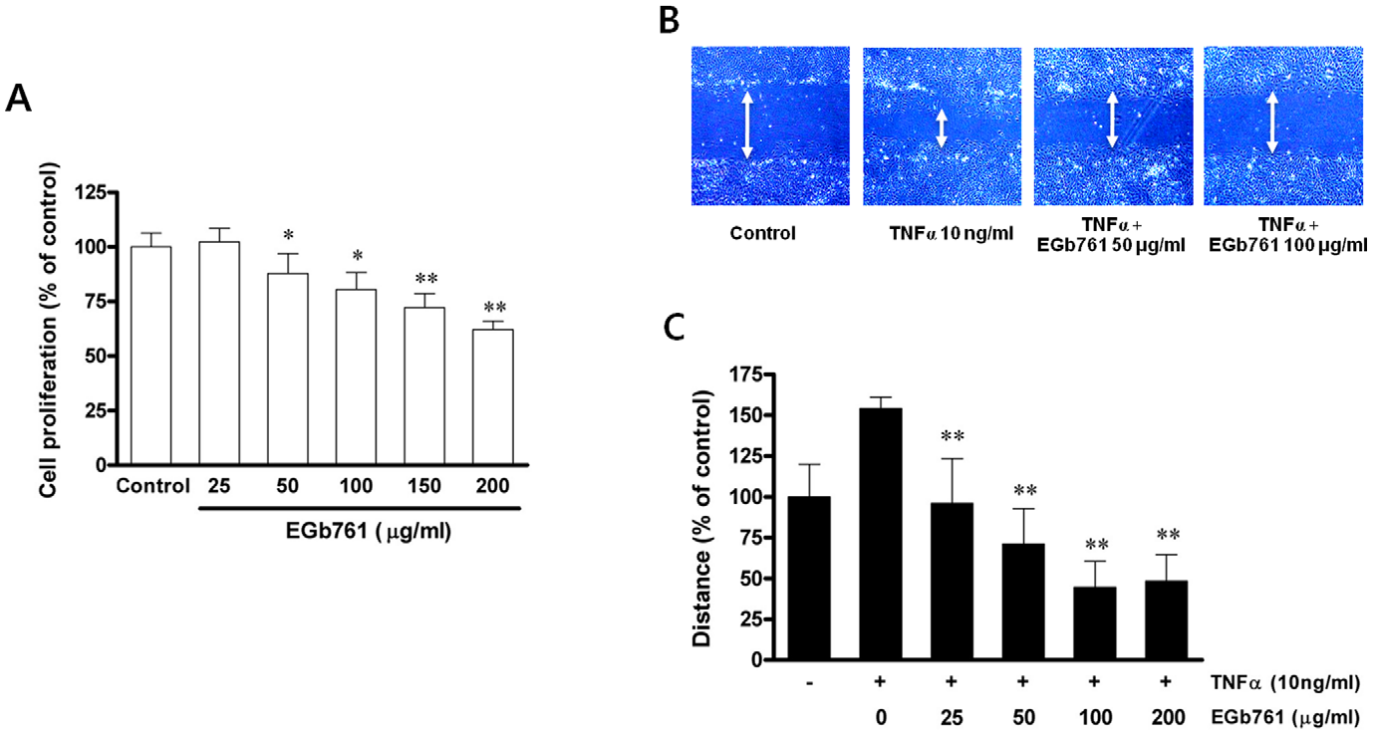
Effects of EGb761 on proliferation and TNFα-stimulated migration of rat aortic smooth muscle cells (RAoSMC). A. In MTT viability assays, cell proliferation was significantly decreased in a dose dependent manner by EGb761 treatment (* p<0.05 and ** p<0.01 compared with untreated RAoSMC). B. Measurement of cell migration assessed by wound healing assay. C. Quantification of the migration distance as a percentage of the control value (** p<0.01 compared with TNFα only treatment).

As shown in **Fig. 3A**, EGb761 treatment inhibited proliferation of RAoSMCs. This effect was initiated at 50 µg/ml of EGb761 and was increased dose-dependently to 200 µg/ml. This anti-proliferative property of EGb761 did not induce cytotoxicity, as shown by the results of the calcein measurement (**Fig. S2**). EGb761 treatment also inhibited platelet-derived growth factor (PDGF)-stimulated RAoSMC proliferation (**Fig. S3**). Furthermore, EGb761 treatment inhibited TNFα-directed migration in rat RAoSMCs dose-dependently (**Fig. 3B, C** **)**. When subcompounds of EGb761 were used, kaemferol and quercetin inhibited TNFα-directed RAoSMC migration while bilobalide showed no change (**Fig. S4**).

### Decrease in Monocyte Adhesion

**Figure 4:**
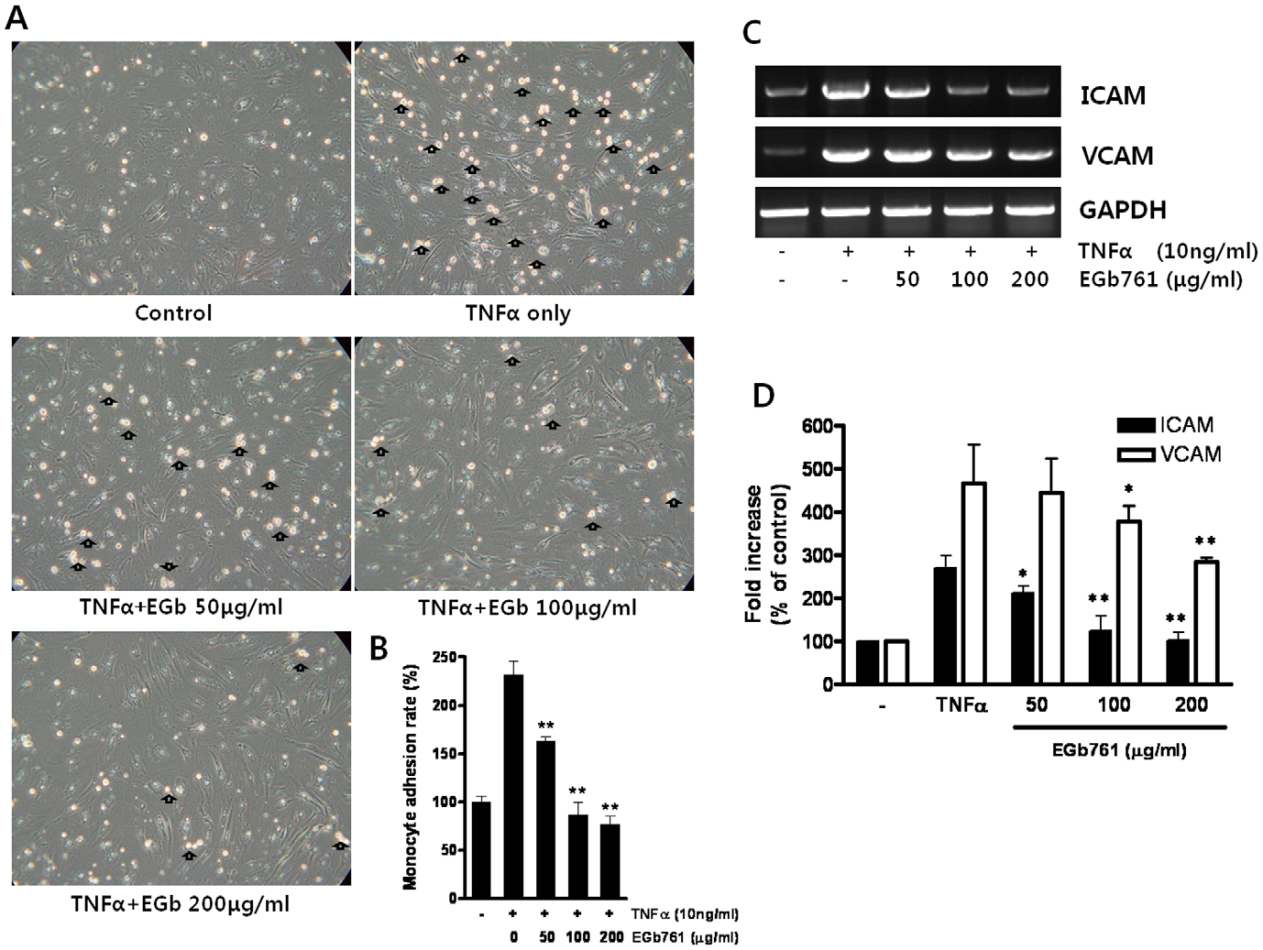
Effect of EGb761 on monocyte adhesion and adhesion molecule expression. A. EGb761 treatment reduced TNFα-stimulated monocyte adhesion using U937 cells (Open arrows indicate adhered monocytes). B. Monocyte adhesion rate as a percentage of the control value (** p<0.01 compared with TNFα only treated group). C and D. ICAM and VCAM expression levels in human umbilical vein endothelial cells. (* p<0.05 and ** p<0.01 compared with TNFα treatment).

The adhesion of inflammatory cells has a critical role in the development of atherosclerosis. Increased monocyte adhesion was observed after TNFα stimulation of HUVECs in the monocyte adhesion assay (**Fig. 4A**). As shown in **Fig. 4B**, EGb761 treatment significantly and dose-dependently decreased monocyte adhesion.

### Effect of EGb761 on ICAM and VCAM Expression in HUVECs

Adhesion molecules such as ICAM and VCAM are also involved in the development of restenosis. In this study, expression of ICAM and VCAM were significantly decreased by treatment with EGb761 at both 100 and 200 µg/ml (**Fig. 4C and 4D** **).**

### Caspase-3 Activity and DNA fragmentation

**Figure 5:**
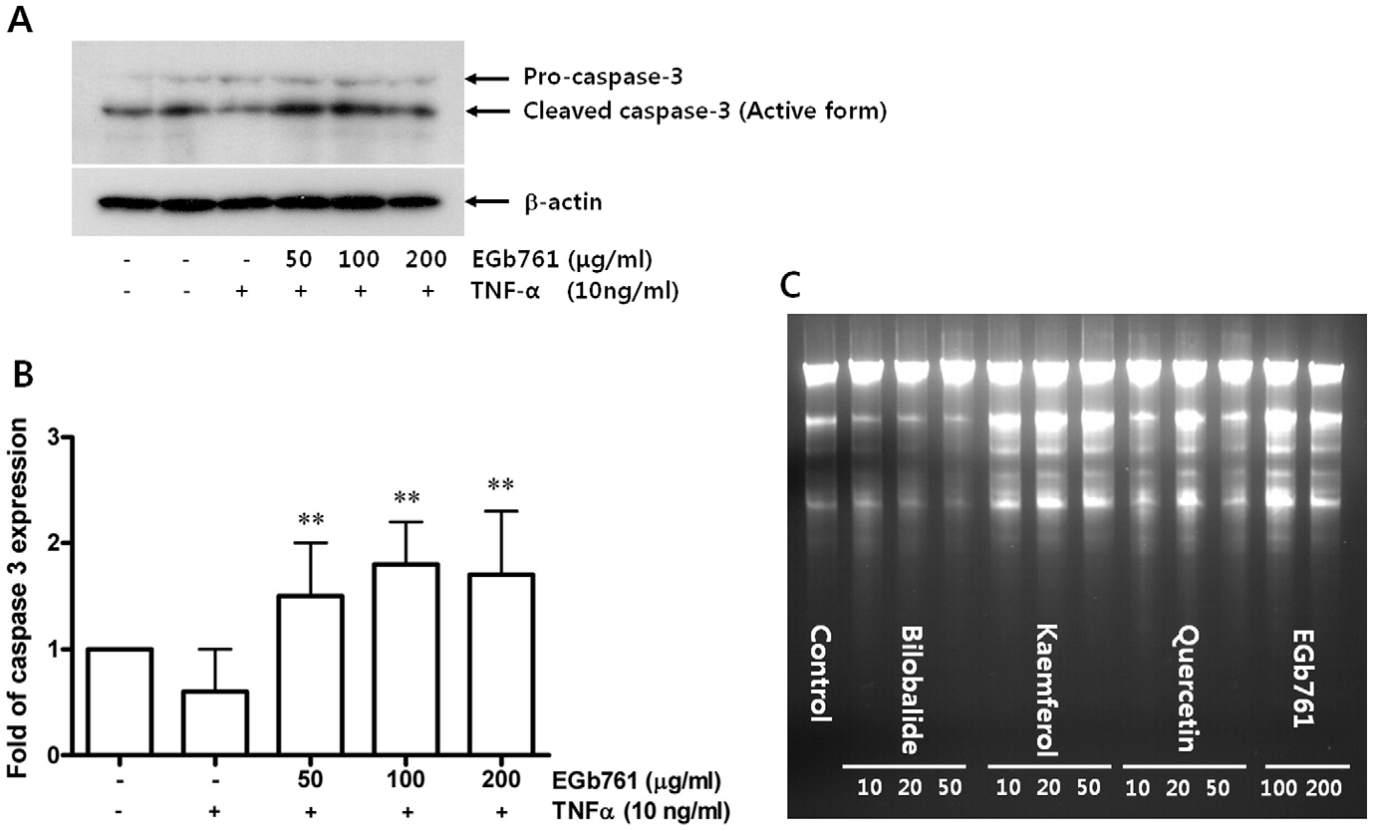
Effect of EGb761 on apoptosis. A. Induction of apoptosis shown by the activation of caspase-3 with EGb761 treatment. B. Dose-dependent increasing pattern of caspase-3 activity by EGb761 (**p<0.05 compared with TNFα only treatment). C. DNA fragmentation by bilobalide, kaemferol, quercetin and EGb761.

Apoptosis of cells is an important contributor to neointimal formation inhibition. To evaluate effect of EGb761 and its subcompounds on apoptosis *in vitro*, caspase-3 activity and DNA fragmentation assay were used. EGb761 treatment increased the level of cleaved caspase-3, reflecting apoptosis in RAoSMCs (**Fig. 5A and 5B**). Treatment of kaemferol or quercetin also increased caspase activity significantly (**Fig. S5**). Treatment of kaemferol, quercetin and EGb761 increased DNA fragmentation compared to control group. Subcompound bilobalide also did not increase DNA fragmentation (**Fig. 5C**).

### Effect of EGb761 Treatment on Glucose Metabolism, Plasma Adiponectin and Inflammatory Markers

After 6 weeks of treatment with EGb761, significant improvement of postload glucose excursion was found in both EGb100 and EGb200 groups compared with control (**Table 1**). Furthermore, EGb761 treatment increased plasma adiponectin concentrations and decreased hsCRP concentrations (**Table 1**). Although the EGb761-treated groups showed lower fasting insulin levels and HOMA-IR than controls, these were not statistically significant. Similarly, the levels of other inflammatory markers such as MCP1, TNFα and PAI1 were lower in EGb761-treated rats than in controls, but these were not statistically significant.

**Table 1:** Weight, Biochemical Parameters, Insulin Resistance Index and Inflammatory Markers in Obese Rat Model of Type 2 Diabetes at the End of 6 Weeks of EGb761 Treatment.

	Control (n = 10)		EGb100 (n = 10)		EGb200 (n = 10)		P Value†
	Mean	SD	Mean	SD	Mean	SD	
Weight, g	583.1	29.3	576.3	27.5	575.5	33.8	
Liver weight, g	3.30	0.65	3.03	0.26	2.98	0.22	
White adipose fat weight, g	1.35	0.21	1.27	0.16	1.29	0.20	
Fasting glucose, mg/dl	119.6	12.6	117.2	8.6	116.2	11.8	
2h postload glucose, mg/dl	204.9	28.0	168.8	27.2	169.4	15.8	A,B
AUC_glucose_	808.5	45.7	659.0	66.3	703.7	66.7	A,B
Fasting insulin, pg/ml	182.6	106.7	177.6	79.9	168.6	90.9	
Total cholesterol, mg/dl	82.6	15.3	81.9	12.9	77.6	9.9	
Triglyceride, mg/dl	60.5	21.9	58.4	26.9	48.7	17.2	
HDL-cholesterol, mg/dl	23.5	4.1	23.2	4.3	22.9	2.2	
LDL-cholesterol, mg/dl	47.0	12.0	47.0	12.5	44.9	8.9	
MCP1, pg/ml	195.5	67.5	160.5	53.0	179.9	75.6	
TNFα, pg/ml	8.5	3.3	6.2	3.2	6.1	2.3	
PAI1, pg/ml	313.1	162.0	212.0	82.9	259.6	108.7	
Adiponectin, µg/ml	10.6	2.4	11.1	3.6	14.5	4.3	A,B
HsCRP, mg/l	0.19	0.04	0.16	0.02	0.15	0.03	A,B
HOMA-IR	55.3	34.2	51.2	24.0	47.4	26.5	
HOMA-B	1162.0	713.4	1228.1	607.5	1230.9	647.0	

Key: AUC_glucose_, Area under the curve of glucose; HOMA-IR, homeostasis model assessment of insulin resistance; HOMA-B, homeostasis model assessment of beta cell function.

† ANOVA with post hoc test (Tukey's-b) was used (A, B and C; mean significant difference between two groups: A = Control vs. EGb100, B = Control vs. EGb200, C = EGb100 vs. EGb200, p<0.05 in all cases).

## Discussion

Six weeks of treatment with EGb761, a standardized *Gingko biloba* extract, produced significantly less neointimal formation in balloon injured carotid arteries than in the control group in a dose-dependent manner (35.5% in the EGb100 group and 69.3% in EGb200) which was accompanied by reduced proliferation and increased apoptosis of vascular smooth muscle cells (VSMCs). EGb761 treatment also showed anti-atherosclerotic effects *in vitro.* It decreased proliferation and migration, and increased apoptosis of VSMCs. It also decreased monocyte adhesion and levels of adhesion molecules in HUVECs. Among subcomponents of EGb761, kaemferol and quercetin played a major role in the prevention of atherosclerosis.

The proliferation and migration of VSMCs are important contributors to neointimal formation after balloon injury [17], [18]. Apoptosis is also important in this process [19]. Therefore, prior efforts to reduce the extent of restenosis have focused on various interventions that reduced the proliferation and migration of VSMCs or of increased their apoptosis [19], [20]. EGb761 has antioxidant, anti-inflammatory and anti-platelet aggregation effects [2], [5]–[7], [9], [10]. In our study, EGb761 also increased caspase-3 activity in VSMCs. It is known that EGb761 has anti-apoptotic properties particularly in neuronal cells. However, EGb761 may also have different effects on cell survival under specific conditions such as target cells and the dosage used. Several studies showed that EGb761 had proapoptotic effects in high turnover state such as cancer [21]–[23]. Thus, EGb761 could have proapoptotic effects on VSMCs in the development of atherosclerosis.

It is well known that infiltration of inflammatory cells occur early after endothelial denudation [24]–[27] and its inhibition is associated with a reduction in medial VSMC proliferation [24]. These data suggest a central role of inflammatory cells in restenosis and provide insights as to how EGb761 might reduce neointimal growth in arteries after balloon injury. Based on previous studies showing a linear relationship between tissue monocyte content and neointimal area [27] and decreased neointimal thickening through blocking early monocyte recruitment by anti-inflammatory drugs [28], inflammatory responses related with monocyte infiltration might aggravate restenosis.

In this study, EGb761 significantly and dose-dependently reduced the levels of adhesion molecules and the degree of monocyte adhesion. These findings could explain the beneficial effects of EGb761 on preventing atherosclerosis as such infiltrations of inflammatory cells promote an atherosclerotic milieu [18], [25], [29].

There have been several reports showing that EGb761 improves glucose homeostasis [30], [31]. A recent study proved that EGb761 induced insulin secretion and that this was mediated by increased intracellular calcium transients [30]. Another group reported that EGb761 ingestion increased plasma insulin levels in response to oral glucose loading in subjects with type 2 diabetes [31]. These data thus suggest that EGb761 enhances pancreatic beta cell function. Consistent with these studies, the AUC_glucose_ calculated from the IPGTT in our study was decreased slightly but significantly in EGb761-treated groups compared with controls. This improved glucose excursion might also contribute to decreased restenosis, although there is no clear explanation for the lack of a dose-dependent response.

Other possible relevant factors affecting the degree of neointimal formation were also evaluated in this study. Circulating levels of adiponectin were increased significantly in EGb761 treatment in a dose-dependent manner. Adiponectin has attracted considerable attention recently as an adipokine that may have critical roles in the development of atherosclerosis [32]. Importantly, low adiponectin level is a risk factor for the subsequent development of cardiovascular diseases [33]–[37]. Adiponectin directly stimulates NO production from endothelium via activation of AMP-activated protein kinase and eNO synthase [38]. Therefore, increasing adiponectin levels are predicted to improve both insulin sensitivity and endothelial function by multiple mechanisms [39]. In this study, there was a negative correlation between adiponectin and TNFα concentration (r = −0.369, P = 0.027), although TNFα levels were not significantly decreased by EGb761 treatment. This data suggests that reducing restenosis by EGb761 treatment may be mediated by increased adiponectin with decreased TNFα level. In addition, hsCRP, an inflammatory marker, was significantly decreased by EGb761 treatment in this study. Many types and levels of association between hsCRP and atherosclerosis or cardiovascular diseases have been suggested [29], [40]–[43]. The associations confirm that atherosclerosis and insulin resistance share a common inflammatory basis by demonstrating that hsCRP has direct harmful effects on vessel walls [29]. An *in vivo* study supported this by showing that the local administration of proinflammatory cytokines impaired endothelium-dependent vascular relaxation [40]. Collectively, hsCRP has proatherogenic and prothrombic properties [44], which include its interaction with LDL-cholesterol [41] and complement-CRP complexes [43], and its capacity to stimulate tissue factor production by macrophages [42]. Furthermore, cytokines produced by adipocytes, such as IL-1, IL-6, and TNFα, stimulate the hepatic synthesis of CRP [45] and modify glucose and lipid metabolism [46]. Thus, the systemic acute phase response might mediate systemic metabolic impairments and induce atherosclerosis.

EGb761 exerts inconsistent effects on lipid metabolism despite its well known beneficial effects on insulin sensitivity. In our study, treatment of EGb761 did not ameliorate lipid profiles. Our finding is consistent with 2 clinical studies showing that EGb761 treatment did not change lipid profiles in subjects with high cardiovascular risk [47], [48]. In contrast, a study involving rats indicated that EGb761 treatment improved lipid profiles [49] and another study reported that *Ginkgo biloba* extracts reduced lipid peroxidation and scavenged lipid radicals *in vivo* [50]. Thus, based on the inconsistency of evidence, further study is needed to clarify the role of EGb761 on lipid metabolism. In this study, effect of EGb761 on blood pressure was also measured. There were no significant differences in systolic blood pressure between angiotensin II only and angiotensin II+EGb761 treated rats, although decreasing trend could be seen in angiotensin II+EGb761-treatment groups (**Fig. S6**).

Recently, Liu F et al observed that *Ginkgo biloba* extract decreased homocysteine-induced intimal thickening after balloon injury in rabbit abdominal aorta [51]. They suggested that the mechanism was possibly associated with the suppression of MMP-9 expression and increased endogenous p21 expression by *Ginkgo biloba* extract. However, the authors did not provide direct effects of *Ginkgo biloba* extract on SMC proliferation or migration *in vivo* or *in vitro*. We therefore used comprehensive methods to investigate the direct mechanism of action of EGb761, various biochemical parameters including adiponectin, hsCRP and other cytokines, monocyte adhesion, apoptosis as well as immunohistochemical staining for proliferating and apoptotic cells in the injured vessels of obese type 2 diabetic animals. In addition, we further investigated the effects of major subcompounds of EGb761 to identify specific components responsible for preventing restenosis.

In conclusion, treatment with EGb761 was found to reduce restenosis in obese rats with type 2 diabetes after balloon injury to the carotid artery. EGb761 significantly suppressed the proliferation and migration of VSMCs, promoted apoptosis and reduced inflammatory processes. In addition, EGb761 showed favorable effects on glucose homeostasis and on adiponectin and hsCRP levels. Among subcomponents of EGb761, kaemferol and quercetin seem to play a major role in the prevention of atherosclerosis. These findings support an emerging role of EGb761 in reducing cardiometabolic risks. Specific intervention studies are needed to confirm the positive effects of EGb761 in type 2 diabetic patients.

## Supporting Information

Figure S1 Immunohistochemical staining of ED-1 in the injured carotid vessel wall I. Arrows indicate ED-1 positive cells in the representative examples. II. Quantification of ED-1 positive cells among control, EGb100 and EGb200.
(TIF)

Figure S2 Effect of EGb761 (a) and its subcompounds (bilobalide, kaemferol and quercetin) (b) on cell survival with calcein measurement.
(TIF)

Figure S3 Effect of EGb761 on PDGF-induced RAoSMC proliferation (** p<0.01 compared with PDGF only treatment).
(TIF)

Figure S4 Effect of EGb 761 subcompounds on migration of RAoSMC by wound-healing assay (a). Quantification of the migration distance as a percentage of the control value (b) (** p<0.01 compared with TNFα only treatment).
(TIF)

Figure S5 Measurement of caspase activity by treatment of subcompound of EGb761 (kaemferol, quercetin, and bilobalide) (**p<0.01 compared with TNFα only treatment).
(TIF)

Figure S6 Effect of EGb761 (200 mg/kg) or candesartan (4 mg/kg) on blood pressure in OLETF rats. Angiotensin II (ATII) was used to increase blood pressure (*p<0.01, ATII only vs. ATII+Candesartan).
(TIF)

Table S1 Component of EGb761.
(DOC)
