# Supplementary figures and images for: EGb761, a Ginkgo Biloba Extract, Is Effective Against Atherosclerosis In Vitro, and in a Rat Model of Type 2 Diabetes
Source: PLoS One. 2011 Jun 2;6(6):e20301. doi: 10.1371/journal.pone.0020301 (PMC3107221; doi:10.1371/journal.pone.0020301)

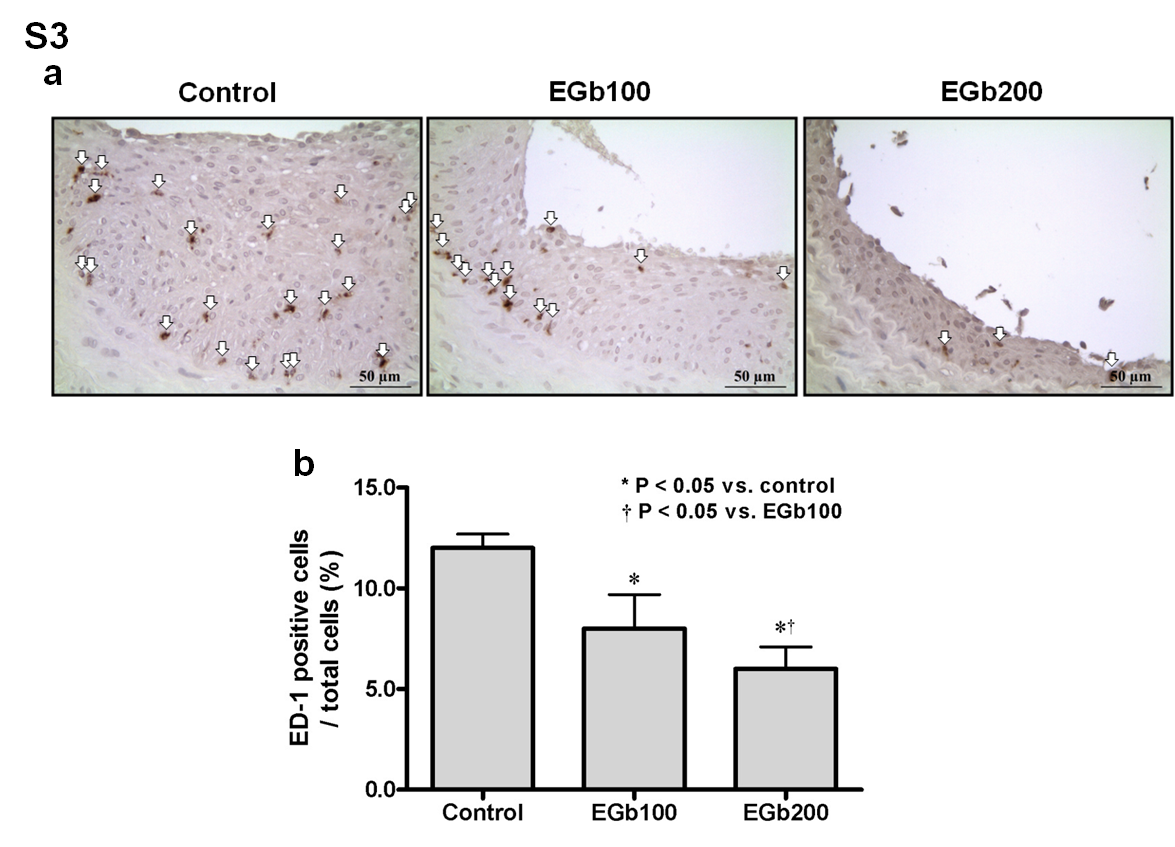

Supplement: Figure S1 — Immunohistochemical staining of ED-1 in the injured carotid vessel wall I. Arrows indicate ED-1 positive cells in the representative examples. II. Quantification of ED-1 positive cells among control, EGb100 and EGb200. (TIF) [file pone.0020301.s001.tif]

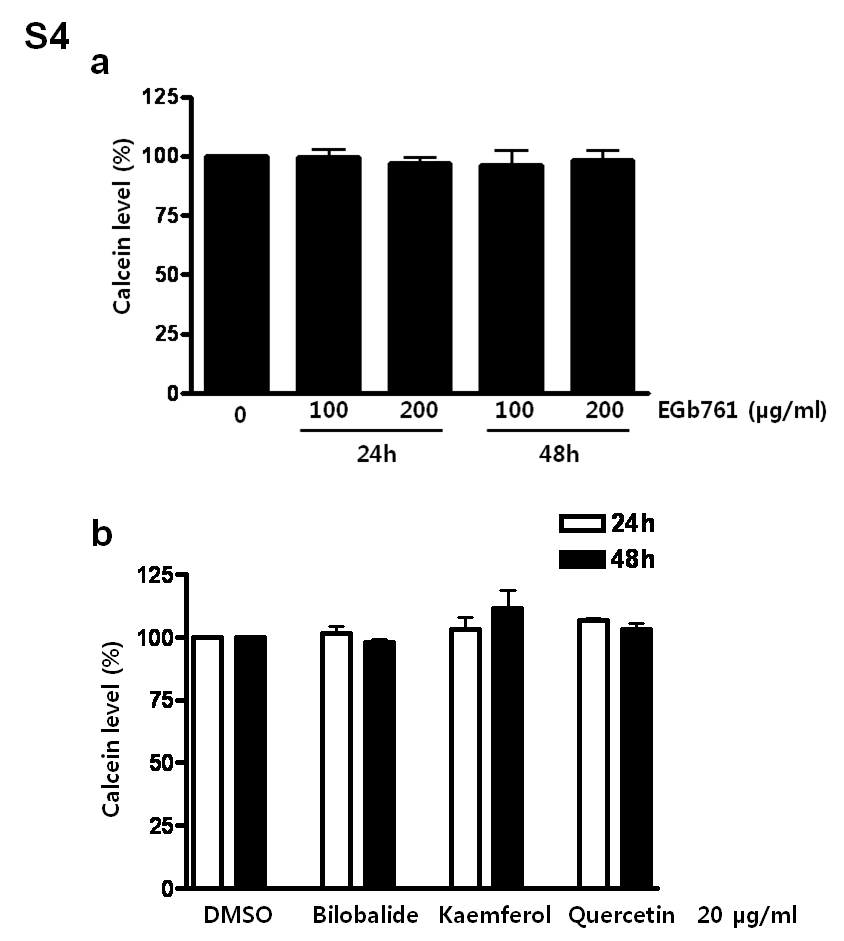

Supplement: Figure S2 — Effect of EGb761 (a) and its subcompounds (bilobalide, kaemferol and quercetin) (b) on cell survival with calcein measurement. (TIF) [file pone.0020301.s002.tif]

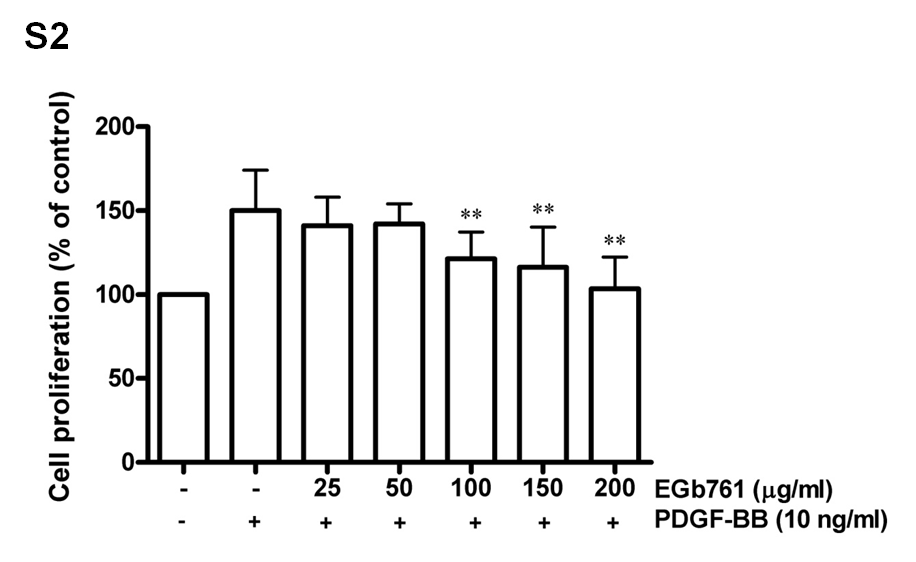

Supplement: Figure S3 — Effect of EGb761 on PDGF-induced RAoSMC proliferation (** p<0.01 compared with PDGF only treatment). (TIF) [file pone.0020301.s003.tif]

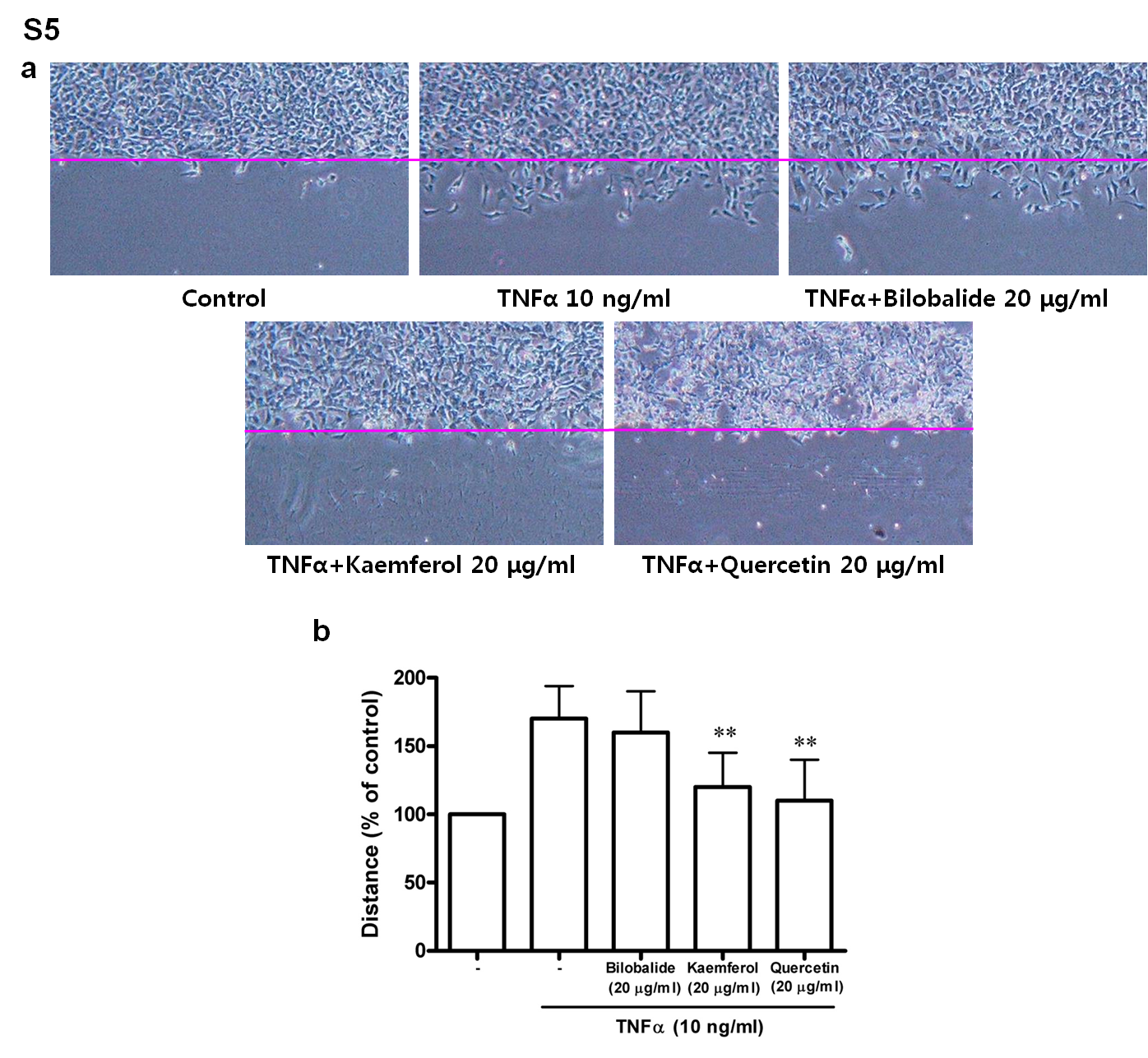

Supplement: Figure S4 — Effect of EGb 761 subcompounds on migration of RAoSMC by wound-healing assay (a). Quantification of the migration distance as a percentage of the control value (b) (** p<0.01 compared with TNFα only treatment). (TIF) [file pone.0020301.s004.tif]

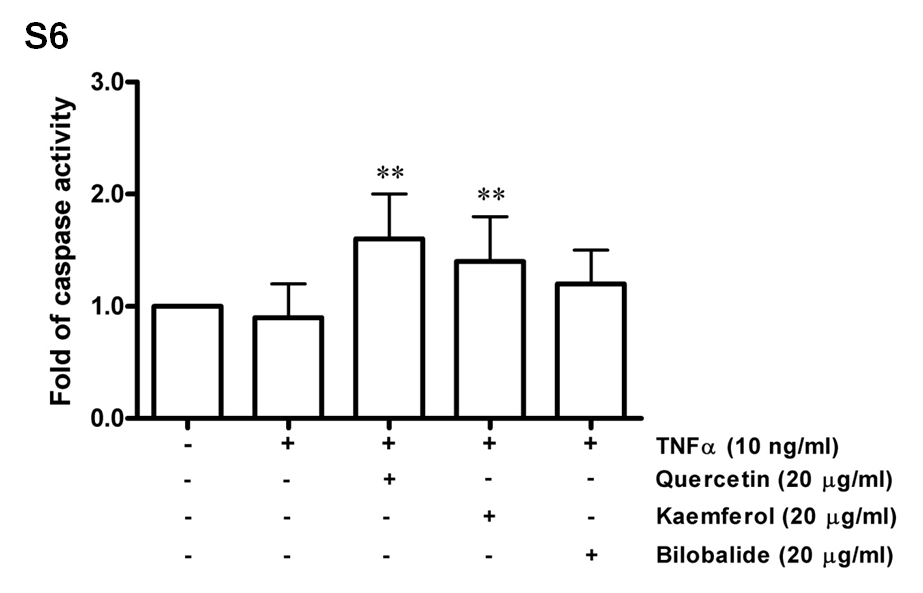

Supplement: Figure S5 — Measurement of caspase activity by treatment of subcompound of EGb761 (kaemferol, quercetin, and bilobalide) (**p<0.01 compared with TNFα only treatment). (TIF) [file pone.0020301.s005.tif]

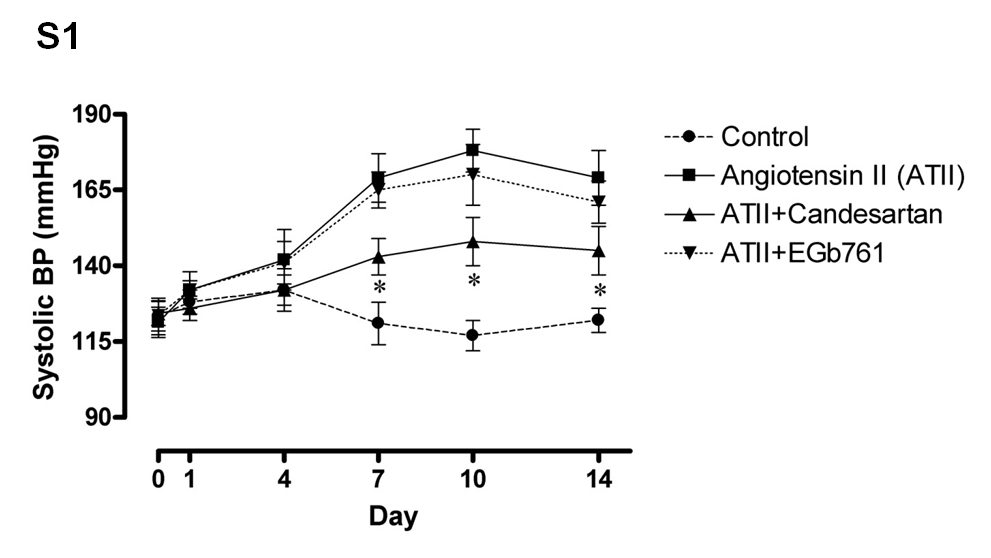

Supplement: Figure S6 — Effect of EGb761 (200 mg/kg) or candesartan (4 mg/kg) on blood pressure in OLETF rats. Angiotensin II (ATII) was used to increase blood pressure (*p<0.01, ATII only vs. ATII+Candesartan). (TIF) [file pone.0020301.s006.tif]
