# Supplementary material for: EGb761, a Ginkgo Biloba Extract, Is Effective Against Atherosclerosis In Vitro, and in a Rat Model of Type 2 Diabetes
Source: PLoS One. 2011 Jun 2;6(6):e20301. doi: 10.1371/journal.pone.0020301 (PMC3107221; doi:10.1371/journal.pone.0020301)
Supplement: Table S1 — Component of EGb761. (DOC) [file pone.0020301.s007.doc]

| **Table S1. Component of EGb761** | | |
| --- | --- | --- |
| **Component** | | **Content (%)** |
| Ginkgo Flavone Glycoside | Quercetin | 11.71 |
|  | Kaemferol | 10.70 |
|  | Isorhamnetin | 2.20 |
|  | total | 24.31 |
| Terpene Trilactones | Bilobalide | 2.65 |
|  | Ginkgolide A | 1.11 |
|  | Ginkgolide B | 0.78 |
|  | Ginkgolide C | 0.88 |
|  | total | 5.42 |
|  |  |  |
| Proanthocyanidines | | 7.0 |
| Carboxlic acid | | 13.0 |
| Catechines | | 2.0 |
| Nonflavone glycoside | | 20.0 |
| Others (high molecules, inorganic, water etc) | | 28.0 |
